# Understanding the coupling of non-metallic heteroatoms to CO_2_ from a Conceptual DFT perspective

**DOI:** 10.1007/s00894-024-05992-3

**Published:** 2024-06-10

**Authors:** Maxime Ferrer, José Elguero, Ibon Alkorta, Luis Miguel Azofra

**Affiliations:** 1 Instituto de Química Médica, CSIC, Juan de La Cierva,3, 28006 Madrid, Spain; 2 PhD Program in Theoretical Chemistry and Computational Modelling, Doctoral School, Universidad Autónoma de Madrid, 28049 Madrid, Spain; 3 Instituto de Estudios Ambientales y Recursos Naturales (iUNAT), Universidad de Las Palmas de Gran Canaria (ULPGC), Campus de Tafira, 35017 Las Palmas de Gran Canaria, Spain

**Keywords:** Amine-CO_2_ coupling, Alcohol-CO_2_ coupling, Thiol-CO_2_ coupling, REG, Density functional theory (DFT)

## Abstract

**Context:**

A Conceptual DFT (CDFT) study has been carry out to analyse the coupling reactions of the simplest amine (CH_3_NH_2_), alcohol (CH_3_OH), and thiol (CH_3_SH) compounds with CO_2_ to form the corresponding adducts CH_3_NHCO_2_H, CH_3_OCO_2_H, and CH_3_SCO_2_H. The reaction mechanism takes place in a single step comprising two chemical events: nucleophilic attack of the non-metallic heteroatoms to CO_2_ followed by hydrogen atom transfer (HAT). According to our calculations, the participation of an additional nucleophilic molecule as HAT assistant entails important decreases in activation electronic energies. In such cases, the formation of a six-membered ring in the transition state (TS) reduces the angular stress with respect to the non-assisted paths, characterised by four-membered ring TSs. Through the analysis of the energy and reaction force profiles along the intrinsic reaction coordinate (IRC), the ratio of structural reorganisation and electronic rearrangement for both activation and relaxation energies has been computed. In addition, the analysis of the electronic chemical potential and reaction electronic flux profiles confirms that the highest electronic activity as well as their changes take place in the TS region. Finally, the distortion/interaction model using an energy decomposition scheme based on the electron density along the reaction coordinate has been carried out and the relative energy gradient (REG) method has been applied to identify the most important components associated to the barriers.

**Methods:**

The theoretical calculation were performed with Gaussian-16 scientific program. The B3LYP-D3(BJ)/aug-cc-pVDZ level was used for optimization of the minima and TSs. IRC calculations has also been carried out connecting the TS with the associated minima. Conceptual-DFT (CDFT) calculations have been carried out with the Eyringpy program and in-house code. The distortion/interaction model along the reaction coordinate have used the decomposition scheme of Mandado et al*.* and the analysis of the importance of each components have been done with the relative energy gradient (REG) method.

**Supplementary Information:**

The online version contains supplementary material available at 10.1007/s00894-024-05992-3.

## Introduction

The increasing concentration of carbon dioxide (CO_2_) in the atmosphere is one of the most important environmental challenges that science faces nowadays. A number of methodologies have been proposed to reduce the amount of CO_2_ in the atmosphere, including geological sequestration [1–3], absorption, adsorption, and membrane technologies [4]. In addition, several organic molecules are capable of react and activate CO_2_ as carbenes [5–9], phosphines [10, 11], frustrated Lewis pairs [12–21], eutectic solvents [22–24], or metal–organic frameworks (MOFs) [25–28], amongst others [29, 30].

The CO_2_ absorption by amine solutions, specially monoethanolamine (MEA) [31–34], is frequently used industrially. Also, supported amines [34–37] and nitrogen heterocycles are capable of react with CO_2 _[38]. In general, this reaction processess with the formation of the carbamic acid followed in some cases by deprotonation to yield the corresponding carbamate [39–41].

Conceptual DFT (CDFT) provides a series of hierarchy chemical concepts that allow an analysis of the chemical reactivity directly associated with physicochemical properties of both global and local nature [42, 43]. Amongst the contributors to this methodology, Prof Toro-Labbé has shown its utility in an important number of chemical reactions [44], including the reinterpretation of the Woodward–Hoffmann rules [45], glycosylation reactions [46, 47], reactivity of hydrogenases [48], reduction of carbon dioxide [49], and expanded its application to the atomic level [50] as well as developing the theory of bond reactivity [51].

In the present article, the reactions of one or two methylamine (CH_3_NH_2_), methanol (CH_3_OH), and methanethiol (CH_3_SH) molecules with CO_2_ have been studied (Scheme 1). The reaction coordinates have been analysed with Conceptual DFT (CDFT) tools. In addition, the strain/interaction model [52–55] coupled with the non-covalent interactions energy decomposition analysis (EDA-NCI) [56, 57] have been analysed with the relative energy gradient (REG) method [58–62] to determine the most important energy contributions to the barriers.

**Scheme** 1. Studied reactions

$${\left({H}_{3}C-XH\right)}_{n}+{C{O}_{2}\rightleftharpoons {\left({H}_{3}C-XH\right)}_{n}}_{-1}+{H}_{3}C-XC{O}_{2}H$$

$$X=N\left(H\right),O,S; n=\text{1,2}$$

## Computational methods

All geometries have been fully optimised, in vacuum, with the hybrid Becke [63], three-parameter, Lee–Yang–Parr [64] density functional (B3LYP) and the Dunning basis set aug-cc-pVDZ [65]. The dispersion has been taken into account by means of the D3 method with the Becke-Johnson damping factor [66], D3(BJ). The synchronous transit-guided quasi-Newton (STQN) method [67] has been used to locate the transition states (TS). Frequency calculations have been carried out in order to verify that the modelled structures correspond to energetic minima or true TSs by the presence of none and one imaginary frequency, respectively. The intrinsic reaction coordinate (IRC) procedure, in which the reaction coordinate, ξ, is expressed in mass-weighted internal coordinates [68], has been used to describe the intermediate structures that connect the three stationary points: reactant, TS, and product. All calculations have been performed thanks to the facilities provided by the Gaussian16 package [69]. The natural bond orbital (NBO) theory [70] has been employed to calculate the electronic population on selected atomic centres using the NBO 3.1 program. The Eyringpy program has been used to calculate the reaction force and the components of the force in the different reaction regimes [71].

The distortion/interaction model [54, 72] has been calculated along the reaction coordinate. The energy at each point of the reaction coordinate is divided into the sum of the interaction energy (E_int_) and the deformation energy (E_def_) (see Eq. 1). The interaction energy is divided, using the energy decomposition scheme proposed by Mandado et al*.*, [56] into electrostatic (E_elec_), Pauli (E_Pauli_) and polarisation (E_pola_) contributions (see Eq. 2). The energy decompositions were obtained using EDA-NCI software [56, 57].

1 $$\Delta E={E}_{int}+{E}_{def}$$

2 $${E}_{int}={E}_{elec}+{E}_{Pauli}+{E}_{pola}$$

The relative energy gradient (REG) method, developed by Thacker et al*.*, [58] has been used to analyse the importance of the different components of the energy in the calculated barriers (Eq. 3). This method compares the variation of the relative energy along the reaction coordinate with the variation of the different EDA components. Linear regressions between each pair of energies provide the Pearson correlation coefficient and the slope of the regression (REG) (Eq. 4). Good Pearson correlation (Eq. 5) indicates that the energy component can explain the overall profile and the value of REG provides its importance to explain it. A detailed description of this method is provided in the original article together with some applications [58–61], including a recent one that uses the energy decomposition scheme of Mandado et al. [62]

3 $${E}_{tot}\left(s\right)={\sum }_{i=1}^{N}{E}_{i}\left(s\right)$$

4 $${E}_{i}\left(s\right)={m}_{REG,i} \cdot {E}_{tot}\left(s\right)+{c}_{i}$$

5 $${R}_{i}=\frac{{\sum }_{s=1}^{M}\left({E}_{i}\left(s\right)-\overline{{E}_{i}}\right)\left({E}_{tot}\left(s\right)-{\overline{E}}_{tot}\right)}{\sqrt{{\sum }_{s=1}^{M}{\left({E}_{i}\left(s\right)-\overline{{E}_{i}}\right)}^{2}}\sqrt{{\sum }_{s=1}^{M}{\left({E}_{tot}\left(s\right)-{\overline{E}}_{tot}\right)}^{2}}}$$

## Results and discussions

To analyse the coupling between non-metallic heteroatoms to CO_2_, the simplest amine, alcohol, and thiol compounds have been selected as model substrates, namely methylamine (CH_3_NH_2_), methanol (CH_3_OH), and methanethiol (CH_3_SH), respectively (Scheme 1). These substrates act as nucleophiles while CO_2_ acts as electrophile for a reaction path comprising the CH_3_XH···CO_2_ complex as reactant, [CH_3_XHCO_2_]^‡^ as TS, and the CH_3_X–CO_2_H adduct as product, where X refers to the heteroatom [X = N(H), O, or S]. In addition, the reaction where a second molecule of the nucleophile assists the reaction has been considered. The reactions with a single nucleophile molecule are denoted as non-assisted while those with two nucleophile molecules are named as assisted along the text.

**Fig. 1:**
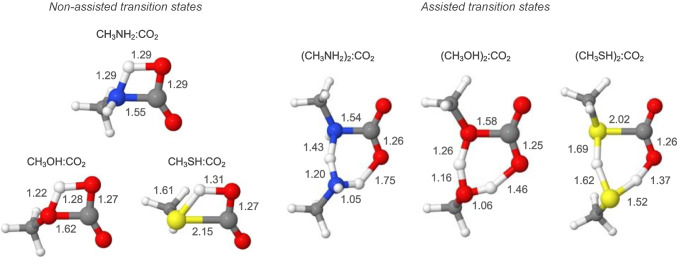
Optimised transitions states for the coupling of CO_2_ with models of amine, alcohol, and thiol substrates. Non-assisted and assisted TSs refer to saddle points in which an additional nucleophilic molecule does not and does act as HAT assistant, respectively. Selected distances are shown in Å. Colour code: yellow, red, blue, grey, and white spheres refer to S, O, N, C, and H atoms, respectively

Figure 1 gathers the optimised structures for the TSs in the non-assisted and assisted reactions. In the non-assisted TSs the incipient X–C is forming and the HAT directly occurs from X to the CO_2_ oxygen atom through a four-membered ring structure. In the assisted TSs the additional nucleophilic molecule acts as a proton bridge: the assistant molecule donates its H atom to CO_2_ and receives a H atom from RXH, leading to the formation of a six-membered ring with lower angular stress. At structural level, this imposes certain characteristics between some TSs and others. First, for the non-assisted cases, the heteroatom-carbon distances for the formation of the incipient X–C bond are longer than those computed for the assisted TSs, being especially remarkable for CH_3_OH and CH_3_SH, 1.62 *vs*. 1.58 Å and 2.15 *vs*. 2.02 Å, respectively, although in a less extent for CH_3_NH_2_, 1.55 *vs*. 1.54 Å. In other words, the incipient X–C bond formation presents interatomic distances closer to the adduct ones in the assisted TSs than in the non-assisted ones. Interestingly, a similar aspect occurs with the geometrical angle for CO_2_. Thus, the OCO angle in the non-assisted TSs presents lower deformations with respect to the angle in CO_2_ than the assisted ones, specifically 137 *vs*. 133º for X = N(H), 144 *vs*. 138º for X = O, and 142 *vs*. 136º for X = S. Thus, OCO angles in the assisted TSs are closer to the OCO angles observed in the product adduct RX–COOH(see details of the optimised Cartesian coordinates provided in the Supporting Information).

**Fig. 2:**
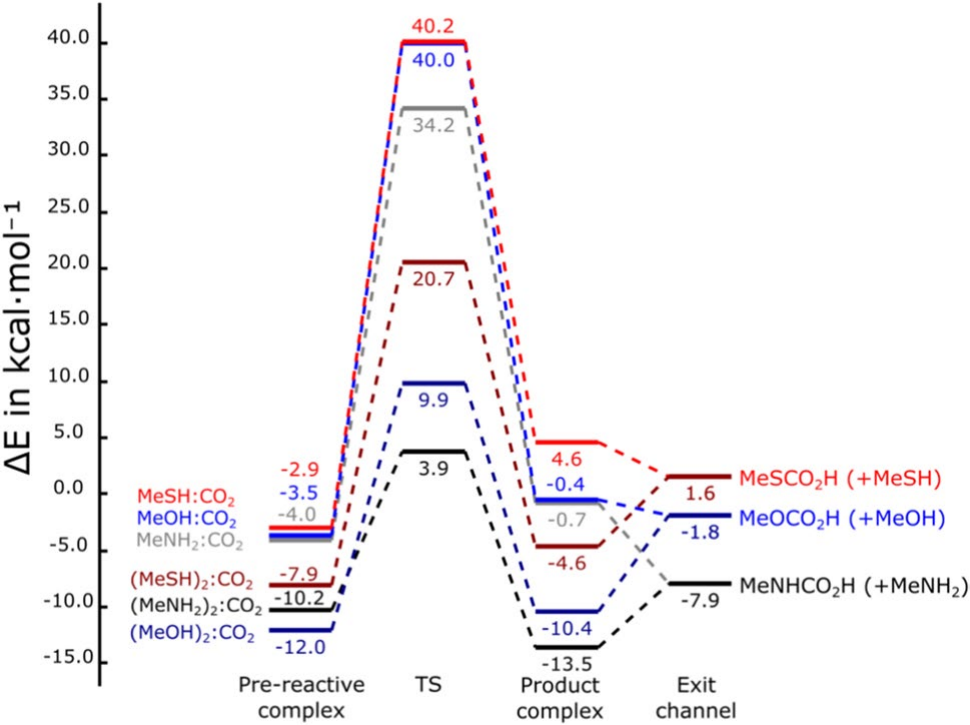
Evolution of the energy of the stationary points of the reactions studied. The 0.0 energy was set as the sum of the energies of the isolated molecules

At energetic level, those TSs which have the participation of an additional nucleophilic molecule as HAT assistant present lower activation energies than those characterising the non-assisted route (Fig. 2). In this sense, the angular stress plays an important role during the formation of the pseudo-cycle in the TS, an effect already observed by us in previous studies when the HAT takes place with the assistance of another molecule, either the same H-donor system (this work) or different such as protic solvent molecules [47, 73].

A comprehensive analysis of the reaction mechanism throughout the entire reaction coordinate is important to understand it. The reaction force, F, defined as the negative derivative of the electronic energy, E, with respect to the reaction coordinate,ξ (Eq. (6)) can be used to analyse a reaction.

6 $$F=-\frac{dE}{d\xi }$$

**Fig. 3:**
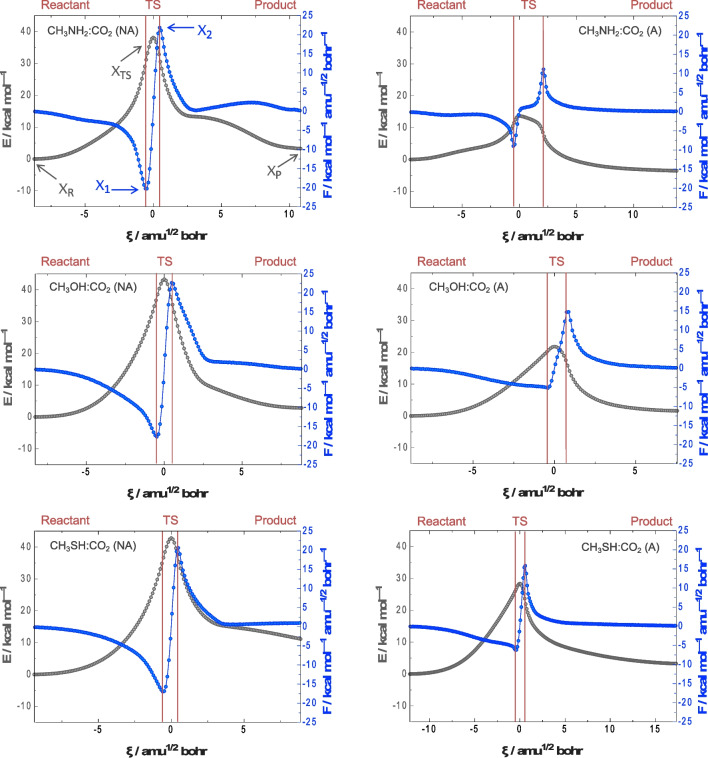
Energy (E, black circles), in kcal mol^–1^, and reaction force (F, blue circles), in kcal mol^–1^ amu^–½^ bohr^–1^, profiles *vs*. the reaction coordinate, ξ, in amu^½^ bohr, for the different coupling reactions between CH_3_NH_2_, CH_3_OH, and CH_3_SH with CO_2_. Reactant, TS, and product regions are delimited by red lines. Note that ‘NA’ and ‘A’ refer to those reaction mechanisms in which an additional nucleophilic molecule does not and does act as HAT assistant/borrower, respectively

Through the representation of both E *vs*. ξ and F *vs*. ξ it can be concluded which is the energy cost for activation and reaction as well as which is the instantaneous rate of change that the energy presents along the path of minimum potential energy in the IRC. Figure 3 gathers the representation of the energy (black) and reaction force (blue) profiles *vs*. the reaction coordinate for the different coupling reactions between CH_3_NH_2_, CH_3_OH, and CH_3_SH with CO_2_ in both non-assisted and assisted cases. These charts, as well as all those presented in this study, are delimited between the same values in order to facilitate a comparative analysis for the different cases. The three critical points of the reaction coordinate ξ_R_, ξ_TS_, and ξ_P_ can be defined as the ones corresponding to the reactant, TS (assigned at 0 amu^½^ bohr in all cases), and product states. In addition, the minimum and the maximum in the reaction force also define two critical points: ξ_1_ and ξ_2_. Those points allow to partition the reaction coordinate into the so-called reactant, TS, and product regions in the ranges [ξ_R_,ξ_1_], [ξ_1_,ξ_2_], and [ξ_2_, ξ_P_], respectively, with ξ_1_ < ξ_TS_ < ξ_2_. In our case, these areas are separated by red lines at Fig. 3.

The studied chemical reactions R–XH···CO_2_ → RX–CO_2_H, present positive reaction electronic energies, E_R_, with the exception of the coupling between methylamine and CO_2_ with a E_R_ computed to be –3.5 kcal mol^–1^. It is worth highlighting that the non-assisted reaction for the coupling between CH_3_SH and CO_2_, E_R_ presents the higher unfavoured value of 11.2 kcal mol^–1^. Interestingly, activation electronic energies, E_ac_, exhibit important decreases when the coupling reactions take place through the formation of six-membered ring TSs, that is when the coupling is assisted. This is especially relevant for the reaction between CH_3_NH_2_ and CO_2_, with a decrease from 38.2 to 13.7 kcal mol^–1^, and in similar but slightly lower extent for CH_3_OH and CH_3_SH with decreases from 43.3 to 21.8 kcal mol^–1^ and 42.8 to 28.4 kcal mol^–1^, respectively. These decreases in activation energy give us an idea about the cost due to angular stress. It is observed that the lower the size of the nucleophilic non-metallic atom, the greater the decrease in activation energy. Specifically, electronic energy drops have been calculated in 24.5, 21.5, and 14.4 kcal mol^–1^ for X = N(H), O, and S, respectively. Although it is not possible to discern what part of the activation energy is due to the formation of the X–C bond and what part is due to HAT, the reaction force allows to determine what part is due to structural reorganisation and what other part is due to electronic rearrangement. To do so, the quantities W_1_ and W_2_, defined as the negative integration of the force in the ranges [ξ_R_, ξ_1_] and [ξ_1_, ξ_TS_], are used, respectively. See eqns. (7) and (8):

7 $${W}_{1}=-{\int }_{{\xi }_{R}}^{{\xi }_{1}}F\left(\xi \right)d\xi >0$$

8 $${W}_{2}=-{\int }_{{\xi }_{1}}^{{\xi }_{TS}}F\left(\xi \right)d\xi >0$$

Note that

9 $${W}_{1}+{W}_{2}={E}_{ac}$$

This partition of the activation energy for the forward path into works W_1_ plus W_2_, Eq. (9), delimited by the instantaneous rate of change in the energy or the decrease/increase of the reaction force profiles, yields interesting results as shown in Table 1. For all cases, it is confirmed that work for structural reorganisation of the system (W_1_), that is, how the system expends energy in preparing for the reactive event, slightly increases in percentage for the assisted processes with respect the non-assisted ones: the participation of an additional nucleophilic molecule as HAT assistant implies greater conformational changes until reaching the reactive intermediate at ξ_1_. The opposite trend occurs for the relaxation energy, that is, the energy released from the TS to the product formation, which would be the activation energy for the reverse path. In analogy with W_1_ and W_2_, the relaxation energy can be also partitioned into W_3_ (electronic) plus W_4_ (structural) [see eqns. (10) and (11)]. The percentage of the W_3_:W_4_ values for the non-assisted and assisted processes are 19:81 *vs*. 33:67 for CH_3_NH_2_ + CO_2_, 19:81 *vs*. 30:70 for CH_3_OH + CO_2_, and in lower extent for CH_3_SH + CO_2_ with 19:81 *vs*. 20:80, *i*.*e*., minimising the work associated to electronic rearrangement, W_3_, towards the formation of the RX–COOH adduct from the TS. As previously observed for electronic energy drops in the activation barriers, the smaller the size, the greater the basicity of the nucleophilic non-metallic atom, and the greater the percentage of electronic rearrangement, W_3_, for the relaxation energy.

**Table 1:** Activation, E_ac_^→^, and reaction, E_R_^→^, energies for the forward paths, and corresponding geometrical (W_1_ and W_4_) and electronic (W_2_ and W_3_) partitions to the energy, all in kcal mol^–1^. In parentheses, percentage of W with respect to the activation energy. Note that both reactant and product refer to adduct structures between the amine, alcohol, or thiol and CO_2_. Note that ‘NA’ and ‘A’ refer to those reaction mechanisms in which an additional nucleophilic molecule does not and does act as HAT assistant/borrower, respectively

System	E_ac_^→^	E_R_^→^	W_1_	W_2_	W_3_	W_4_
CH_3_NH_2_:CO_2_ (NA)	38.2	3.3	31.3 (82)	6.9 (18)	–6.7 (19)	–28.2 (81)
(CH_3_NH_2_)_2_:CO_2_ (A)	13.7	–3.5	11.3 (83)	2.3 (17)	–5.6 (33)	–11.6 (67)
CH_3_OH:CO_2_ (NA)	43.3	2.8	37.7 (87)	5.6 (13)	–7.6 (19)	–32.9 (81)
(CH_3_OH)_2_:CO_2_ (A)	21.8	1.6	20.4 (94)	1.4 (6)	–6.0 (30)	–14.2 (70)
CH_3_SH:CO_2_ (NA)	42.8	11.2	36.5 (85)	6.3 (15)	–6.0 (19)	–25.6 (81)
(CH_3_SH)_2_:CO_2_ (A)	28.4	3.3	26.6 (94)	1.7 (6)	–5.1 (20)	–20.0 (80)

10 $${W}_{3}={\int }_{{\xi }_{TS}}^{{\xi }_{2}}F\left(\xi \right)d\xi <0$$

11 $${W}_{4}={\int }_{{\xi }_{2}}^{{\xi }_{P}}F\left(\xi \right)d\xi <0$$

**Fig. 4:**
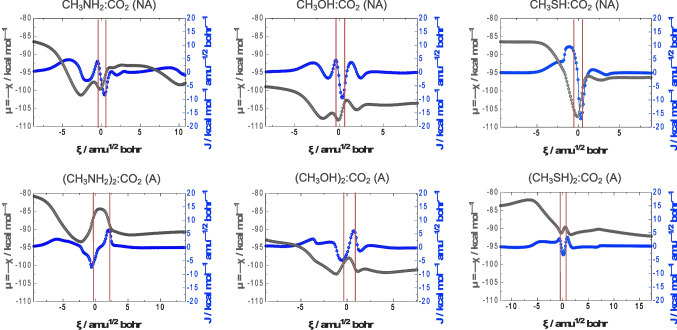
Electronic chemical potential (μ, black circles; equal to the negative of electronegativity, χ), in kcal mol^–1^, and reaction electronic flux or REF (J, blue circles), in kcal mol^–1^ amu^–½^ bohr^–1^, profiles *vs*. the reaction coordinate, ξ, in amu^½^ bohr, for the different coupling reactions between CH_3_NH_2_, CH_3_OH, and CH_3_SH with CO_2_. Reactant, TS, and product regions are delimited by red lines as indicated in Fig. 2. Note that ‘NA’ and ‘A’ refer to those reaction mechanisms in which an additional nucleophilic molecule does not and does act as hydrogen atom transfer assistant, respectively

At Fig. 4, the representation of the electronic chemical potential (black) and reaction electronic flux (blue) profiles *vs*. the reaction coordinate are also shown. Amongst the theoretical tools that Conceptual DFT (CDFT) offers, [43, 74] the electronic chemical potential, μ, which is equal to the negative of the electronegativity, χ, allowing to understand changes at the electronic level directly associated with physicochemical properties of the entities that are involved in each reaction. For a system with N electrons, μ is defined as the first derivative of the electronic energy, E, with respect to N when the external potential, *v*(**r**), remains constant. Given that N is a discontinuous variable, Eq. (12) shows the expression of μ in terms of the lowest unoccupied (LUMO) and highest occupied molecular orbitals (HOMO), ε_L_ and ε_H_. This expression has been obtained by application of finite differences and the Koopmans’ theorem [75] as the negative semi-sum of the first ionisation potential, I, and the electron affinity, A.

12 $$\mu =-\chi ={\left(\frac{\partial E}{\partial N}\right)}_{v\left(\overrightarrow{r}\right)}\approx -\left(\frac{I+A}{2}\right)\approx \frac{{\varepsilon }_{L}+{\varepsilon }_{H}}{2}$$

In similitude with E and F, the reaction electronic flux, J, also known in the CDFT literature by its acronym, REF, is defined as the negative derivative of the electronic chemical potential, μ, with respect to the reaction coordinate, ξ. See Eq. (13):

13 $$J=-\frac{d\mu }{d\xi }$$

In all cases, we find a direct correspondence with what has been observed in the reaction force profiles. At the extremes of the so-called reactant and product zones, where structural reorganisation primally takes place giving sense to the works W_1_ and W_4_, the electronic fluxes are close to the zero regime, that is, an absence of electronic activity. In addition, the most notorious changes (appearance of minima and maxima) in the REF profiles coincide with the limits of the reaction coordinate that delimit the TS region, that is, it is found that the greatest electronic activity appears in those areas close to the saddle point. By analogy with classical thermodynamics, positive values of REF are associated with spontaneous rearrangements of the electron density, the latter being driven by bond strengthening or bond forming processes, while negative values of REF suggest non-spontaneous rearrangements of the electron density that are mainly driven by bond weakening or bond breaking processes [76]. For the case of the reaction between CH_3_NH_2_ and CO_2_, as illustrative example of a non-assisted process, four sections can be discriminated describing the following events: i) from ξ_R_ to *ca*. –3 amu^½^ bohr (positive REF), the approach between the N and C atoms in view of the nucleophilic attack of the former against the latter is observed, resulting in the formation of the dative N → C bond, also accompanied by the bending of the CO_2_ moiety; ii) from *ca*. –3 to –1 amu^½^ bohr (negative REF), a weakening of the N–H bond occurs, preparing the proton for its subsequent transfer; iii) from *ca*. –1 to 1 amu^½^ bohr, the system is immersed in the so-called TS region, observation of a maximum and a minimum at points very close to ξ_1_ and ξ_2_, respectively, associated with the breaking of the aforementioned N–H bond while the O–H bond is being reinforced/formed; iv) from *ca*. ξ_2_ to ξ_P_, REF primally stays in a zero flux regime, that is, the system is structurally reorganising itself for the formation of the lowest energy adduct state RX–COOH. This is also observed for the case of the reaction between CH_3_OH and CO_2_, but also for CH_3_SH and CO_2_ although the weakening of the X–H bond is not as marked as in the two previous cases in the evolution of the REF. For the assisted process in the reaction between CH_3_NH_2_ and CO_2_, also as illustrative example, REF evolves towards positive values along the first steps of the IRC associated to the formation of the dative N → C bond, however, at critical points ξ_1_ and ξ_2_ a minimum and a maximum are observed, that is different to what has been described for the non-assisted cases. Although all these reactions take place in a single step, in the assisted processes the HAT does not occur synchronously. First the nucleophilic CH_3_NH_2_ molecule transfers a H atom to the bridging CH_3_NH_2_ molecule, *i*.*e*., during the decreasing to increasing trend around the minimum at ξ_1_ one N–H bond is breaking while other is forming. Secondly, the assistant CH_3_NH_2_ molecule transfers a H atom to CO_2_, *i*.*e*., during the increasing to decreasing trend around the minimum at ξ_2_ one N–H bond is breaking while one O–H bond is forming. In what concern to the electronic chemical potential, μ, it should be noted that, apart from small fluctuations along the IRC, all systems evolve towards more negative values from reactants to products. This property could be correlated with the corresponding thermodynamic quantity measuring the escaping tendency of an electron [77], therefore evolving to more spontaneous values in the studied examples. However, this interpretation is not exempt from debate within the field [78].

The distortion/interaction model in combination with the non-covalent interaction energy decomposition analysis (EDA-NCI) have been used to quantify four components of the energy (geometry distortion energy, electrostatic, Pauli repulsion, and polarisation) along the reaction. Each reaction is divided into two parts: from the pre-reactive complex to the TS and from the TS to the product. For each of these parts, the linear correlation of each energy component *vs*. the relative energy along the reaction coordinate provides the values of REG (slope of the correlation) for each one. Large and positive values of REG indicate that the corresponding energy component follows the same trend as the total energy along the reaction coordinate while negative values indicate that the energy component opposes to the overall energetic trend. Table 2 shows the REG values for the two parts of the reactions. With few exceptions, the REG values are larger in absolute value for each nucleophile and energy component in the assisted reactions than in the non-assisted ones.

**Table 2:** REG and Pearson correlation coefficient (in parenthesis) values of the four energy components for all the reaction studied. The most important component is highlighted in bold

	(MeXH)_*n*_:CO_2_ → TS				TS → MeX–CO_2_H + (MeXH)_*n*–1_			
Reaction	E_def_	E_elec_	E_Pauli_	E_pola_	E_def_	E_elec_	E_Pauli_	E_pola_
MeNH_2_:CO_2_	1.62 (0.99)	–5.12 (0.98)	**11.21** (0.99)	–6.72 (0.99)	–4.27 (0.95)	3.67 (0.99)	–9.11 (0.98)	**10.71** (0.99)
MeOH:CO_2_	0.93 (0.94)	–2.76 (0.99)	**6.17** (0.99)	–3.34 (0.97)	–3.94 (0.99)	3.76 (0.96)	–9.27 (0.99)	**10.45** (0.99)
MeSH:CO_2_	0.80 (0.96)	–1.82 (0.99)	**4.46** (0.99)	–2.44 (0.96)	–4.03 (0.99)	3.67 (0.99)	–10.16 (0.99)	**11.52** (0.99)
(MeNH_2_)_2_:CO_2_	4.91 (0.99)	–13.23 (0.98)	**24.83** (0.98)	-15.52 (0.98)	–5.56 (0.97)	4.51 (0.93)	–17.03 (0.97)	**19.08** (0.98)
(MeOH)_2_:CO_2_	2.07 (0.92)	–5.12 (0.98)	**9.85** (0.98)	–5.79 (0.96)	–5.24 (0.99)	5.49 (0.98)	–16.26 (0.99)	**17.00** (0.99)
(MeSH)_2_:CO_2_	1.52 (0.93)	–3.00 (0.98)	**6.30** (0.98)	–3.82 (0.94)	–2.53 (0.92)	2.77 (0.90)	–9.84 (0.95)	**10.60** (0.96)

In the first part of the reaction (from the pre-reactive complex to the TS), the dominant component is the Pauli repulsion. This result agrees with the recent publications on the importance of the Pauli repulsion in reactivity [79, 80]. In contrast, the second part of the reaction (from the TS to the adducts) is dominated by the polarisation that shows larger REG values that the absolute values of the Pauli repulsion.

## Conclusions

The reactions of the addition of CO_2_ to methylamine (CH_3_NH_2_), methanol (CH_3_OH), and methanethiol (CH_3_SH) have been theoretically studied at DFT level. The reaction evolves in a single step with simultaneous formation of the heteroatom-carbon bond and hydrogen atom transfer (HAT) from XH [X = N(H), O, S] towards one of the oxygen atoms of CO_2_. The presence of a second nucleophilic molecule assisting in the reactions decreases the reaction barrier due to the six-membered ring formed in the transition state (TS) in contrast to the four-membered ring in the 1:1 reactions. The reaction profiles have been analysed within the framework of the Conceptual DFT approach. Through the analysis of the energy and reaction force profiles along the intrinsic reaction coordinate (IRC), the ratio of structural reorganisation and electronic rearrangement for both activation and relaxation energies has been computed. In this regard, in the assisted processes, *i*.*e*., those reactions in which an additional nucleophilic molecule acts as HAT assistant/borrower, the work associated to electronic rearrangement is minimised in the formation of the RX–COOH adduct from the TS. In addition, the analysis of the electronic chemical potential and reaction electronic flux profiles confirms that the highest electronic activity as well as their changes take place in the TS region. Besides, the distortion/interaction model combined with an energy decomposition method have been used to analyse the most important contributions to these reactions. The application of the relative energy gradient (REG) method in the reaction before and after the TS shows that the Pauli repulsion energy component is dominant from the pre-reactive complex to the TS while polarisation became more important from the TS towards the formation of the adducts.

### Supplementary Information

Below is the link to the electronic supplementary material.

Supplementary file1 (DOCX 23 KB)

## Data Availability

Data is provided within the manuscript or supplementary information files.
