# Supplementary material for: Understanding the coupling of non-metallic heteroatoms to CO2 from a Conceptual DFT perspective
Source: J Mol Model. 2024 Jun 10;30(7):201. doi: 10.1007/s00894-024-05992-3 (PMC11162977; doi:10.1007/s00894-024-05992-3)
Supplement: Supplementary file 1 — Supplementary file1 (DOCX 23 KB) [file 894_2024_5992_MOESM1_ESM.docx]

***Supplementary Data***

***Journal of Molecular Modelling***

**Understanding the coupling of non-metallic heteroatoms to CO_2_ from a Conceptual DFT perspective**

Maxime Ferrer,^1,2^ José Elguero,^1^ Ibon Alkorta,^1,^* and Luis Miguel Azofra^3,^*

^1^ Instituto de Química Médica, CSIC, Juan de la Cierva, 3, E-28006 Madrid, Spain

^2^ PhD Program in Theoretical Chemistry and Computational Modelling, Doctoral School, Universidad Autónoma de Madrid, 28049 Madrid, Spain

^3^ Instituto de Estudios Ambientales y Recursos Naturales (iUNAT), Universidad de Las Palmas de Gran Canaria (ULPGC), Campus de Tafira, 35017 Las Palmas de Gran Canaria, Spain

*Corresponding authors: [ibon@iqm.csic.es](mailto:ibon@iqm.csic.es); [luismiguel.azofra@ulpgc.es](mailto:luismiguel.azofra@ulpgc.es)

Index

S2-7 Optimized geometry (Å) and electronic energy (Hartree) of the stationary points.

Optimized geometry (Å) and electronic energy (Hartree) of the stationary points.

MeNH2:CO2

Total Energy= -284.500489619

NIMAG= 0

C,-2.1397934135,0.7523112476,-0.0959904252

H,-1.7588260924,1.3805778052,-0.91136047

H,-1.890850615,1.25868535,0.8454222466

N,-1.4578706806,-0.5472974474,-0.1349714098

H,-1.7837284599,-1.1502163917,0.6166566006

H,-1.6625913367,-1.0380050247,-1.002114154

C,1.3207382381,-0.1095474456,0.1031727424

O,1.5824755954,-1.2462790401,0.0448864647

O,1.1415721303,1.0431954558,0.1688023487

H,-3.2390653658,0.6998764909,-0.182200944

MeNH2/CO2 (TS)

Total Energy= -284.439571096

NIMAG= 1

C,-1.4006224285,0.5205736906,0.0616204704

H,-1.2457045407,1.312075134,-0.6838837618

H,-1.2376004806,0.9359664726,1.0626131277

N,-0.4424660465,-0.5766273261,-0.1542095559

H,0.1558970803,-1.2772356826,0.7530470521

H,-0.6171493126,-1.0516599162,-1.0405023117

C,1.0549999208,-0.1711432251,-0.058863288

O,1.4121064487,-1.0113606856,0.8564307727

O,1.5738418965,0.7056971244,-0.6955669336

H,-2.4252335475,0.1382705539,-0.0067433818

MeNHCO2H

Total Energy= -284.506690996

NIMAG= 0

C,-1.8210423416,0.7324717817,-0.3062713771

H,-1.2986532801,1.3715542236,-1.0244515533

H,-2.3035171847,1.3660361617,0.4511688138

N,-0.8387037625,-0.1472224198,0.3000206443

H,2.0990318317,-0.9520568471,0.3967416422

H,-1.1206005962,-0.8203591788,0.9966894445

C,0.4755039597,-0.1092299851,-0.0310358079

O,1.1794826235,-1.03976759,0.6859134265

O,0.9965536786,0.6331511421,-0.8463362976

H,-2.5926952084,0.1561992317,-0.8357660153

(MeNH2)2:CO2

Total Energy= -380.388650929

NIMAG= 0

C,-2.0388430972,1.3796571835,-0.4049629086

H,-1.8228777737,2.2455952402,-1.0455171115

H,-1.7543177303,1.6524044619,0.6209277987

N,-1.2321154527,0.2344168603,-0.8346185908

H,-1.3631557749,-0.5603313732,-0.2039695488

H,-1.5042820771,-0.0626596572,-1.7677981142

C,1.3656751512,1.0233186539,-0.3122116981

O,1.537688379,0.1433957147,0.4399172052

O,1.2966619265,1.9381127065,-1.0329803212

N,-0.7093517222,-2.0516963772,1.1765511716

H,-0.0004962108,-1.4257902163,1.551890366

C,-0.0900872131,-3.0039910289,0.2453192388

H,0.6895455415,-3.6442348011,0.6926666858

H,0.3654748518,-2.4432322869,-0.5807671413

H,-1.126608283,-2.5378690125,1.9658290386

H,-3.129970212,1.2069801712,-0.4148295425

H,-0.867012303,-3.654601239,-0.1781175278

(MeNH2)2/CO2 (TS)

Total Energy= -380.366169719

NIMAG= 1

C,-1.4667774171,0.6087678066,-0.0568587387

H,-1.2410873611,1.504648313,-0.6478292839

H,-1.6452570723,0.9291304024,0.9753283576

N,-0.3488531922,-0.3456769949,-0.0741784245

H,-0.348749417,-1.497193557,0.7783789814

H,-0.2096974198,-0.7177149849,-1.0131952139

C,1.0166449833,0.2298493804,0.3564669467

O,1.888241236,-0.6806404666,0.4056138162

O,1.0338164212,1.4255285854,0.6215297426

N,0.1453115876,-2.3913605926,1.4034950077

H,1.1118717254,-2.0224011213,1.221822266

C,-0.0804803699,-3.7258837343,0.8211780856

H,0.5497876698,-4.4834964167,1.3018001615

H,0.1729797704,-3.6819451382,-0.2439725636

H,-0.0604702717,-2.3520337888,2.3991484078

H,-2.370959663,0.120950213,-0.4450454861

H,-1.1340773197,-4.0093360155,0.9265253575

MeNH2:MeNHCO2H

Total Energy= -380.393841988

NIMAG= 0

C,-1.6684053394,1.5063208313,-1.0806087307

H,-1.4036157716,1.5270470058,-2.1497186656

H,-1.630346794,2.5325563007,-0.7015428601

N,-0.7631216558,0.6801963165,-0.2980702164

H,-0.3483540569,-2.2453257663,2.0072811301

H,-0.9247612195,-0.3154181227,-0.2808873521

C,0.5103999369,1.1285744269,-0.0139313323

O,1.3172664673,0.2182107053,0.5735809227

O,0.8828235529,2.262465811,-0.2484748868

N,0.1865138577,-2.2826186662,1.1420866099

H,0.8474850609,-0.6377625688,0.7517485479

C,-0.5457774102,-3.0430370051,0.1138336824

H,0.0294977736,-3.0297274288,-0.8201809993

H,-1.5123205523,-2.557447834,-0.0761525064

H,1.0589406047,-2.7546545959,1.3722415964

H,-2.6887117826,1.1199210206,-0.9695278452

H,-0.7428786716,-4.0911794301,0.3876109055

MeOH:CO2

Total Energy= -304.367007996

NIMAG= 0

C,-2.1518535614,0.7699032022,0.0485686849

H,-1.5254866957,1.5101229089,-0.4613299967

H,-2.6203180464,1.2446681298,0.9253686016

O,-1.2831465071,-0.2995452911,0.4268793444

H,-1.7918679249,-0.979535315,0.8819229245

C,1.3829450421,-0.1938640671,-0.1645550847

O,1.6063407656,-1.2285361625,0.3263601551

O,1.207491214,0.8454743849,-0.6675302122

H,-2.9377172861,0.4312512098,-0.6452324169

MeOH/CO2 (TS)

Total Energy= -304.297553974

NIMAG= 1

C,-1.3964485019,0.5121728214,0.0891699281

H,-1.1845229115,1.2788612939,-0.6659286121

H,-1.2997854468,0.9397968762,1.096883643

O,-0.4583261509,-0.5605233597,-0.1028630974

H,0.1308599698,-1.1920166317,0.7602904584

C,1.1098211111,-0.1721624023,-0.0616382004

O,1.4000712057,-1.0447669908,0.8123287922

O,1.5529474771,0.6977813734,-0.7304336964

H,-2.4032322827,0.1074954496,-0.0590746254

MeOCO2H

Total Energy= -304.362037027

NIMAG= 0

C,-1.8190623229,-0.0781142617,0.

H,-1.9384765051,0.5420914095,-0.8957990641

H,-1.9384765051,0.5420914095,0.8957990641

O,-0.5195410746,-0.7041272957,0.

C,0.525680934,0.1558918703,0.

O,1.6894668701,-0.5211448512,0.

O,0.4530924683,1.3564856915,0.

H,1.500435283,-1.4709063839,0.

H,-2.5366419685,-0.9029028679,0.

(MeOH)2:CO2

Total Energy= -420.125973756

NIMAG= 0

C,-2.1783101105,1.2728407928,-0.3228841912

H,-2.0470014387,2.1697993351,-0.9403020536

H,-2.2645362466,1.5844944019,0.7314236733

O,-1.0457819582,0.4413212796,-0.5410042725

H,-1.0893028451,-0.3410819091,0.0391938717

C,1.5209882066,1.1984753575,-0.1875806059

O,1.7751092413,0.1512599775,0.2715130715

O,1.3438368558,2.259547727,-0.6313226039

O,-0.4303544085,-1.77846991,1.0269073801

H,0.4526457764,-1.3840130356,1.0425507153

C,-0.3780358225,-2.9798138028,0.2491327683

H,0.2904946328,-3.7237058717,0.707597419

H,-0.0509509167,-2.7788099986,-0.7825371473

H,-3.1152152997,0.7754030231,-0.6233145666

H,-1.3954146663,-3.3862013669,0.226630542

(MeOH)2/CO2 (TS)

Total Energy= -420.091094398

NIMAG= 1

C,-1.4682915751,0.5520414001,0.0736961818

H,-1.0983807051,1.3713513685,-0.5498634492

H,-1.7592886715,0.9394089416,1.0597064964

O,-0.422311843,-0.4198008308,0.2031185273

H,-0.4678352442,-1.4800432679,0.8792095762

C,1.0575804586,0.1125437478,0.3084247278

O,1.7632514363,-0.7972053779,0.7947023242

O,1.2044815798,1.2340081778,-0.0954803142

O,0.0067860017,-2.3681618322,1.4586000353

H,0.9343173844,-1.8806820181,1.3143500052

C,-0.0354963996,-3.6009043669,0.7055577783

H,0.632963107,-4.3237381293,1.1850085606

H,0.2793906982,-3.427034174,-0.3320714571

H,-2.3213581234,0.0609913534,-0.4086973858

H,-1.0638049743,-3.9755516119,0.7313495932

MeOH:MeOCO2H

Total Energy= -420.123367229

NIMAG= 0

C,-1.6579944456,1.6348686247,-0.6207547384

H,-1.4772427765,1.8162446134,-1.6864463192

H,-1.5421642923,2.5739698338,-0.0679521059

O,-0.7376000684,0.6447832065,-0.1197781667

H,-1.030011731,-1.3854807883,0.7664662839

C,0.5844315444,0.9958654526,-0.2215649843

O,1.3748782276,0.0468215387,0.2665768963

O,0.9665968608,2.0356494103,-0.6975153649

O,-0.2825964156,-1.9394260397,1.0331982053

H,0.8414581788,-0.7183486039,0.6051755774

C,-0.2459060919,-3.11251386,0.2073508978

H,0.6369943955,-3.6838710226,0.5128606588

H,-0.1550140129,-2.8517737663,-0.8578860758

H,-2.6549573995,1.2132510187,-0.4626477006

H,-1.1423799733,-3.7297426178,0.3616479363

MeSH:CO2

Total Energy= -627.352199911

NIMAG= 0

C,-1.9435999352,0.884752318,-0.0101024722

H,-1.7096871303,1.4848811415,-0.8965852562

H,-1.4399789569,1.3261335953,0.8555629058

S,-1.2980203059,-0.8026042236,-0.3567833953

H,-1.6483274804,-1.3652437313,0.8264574059

C,1.7882035424,0.1556489875,0.4820647409

O,2.2444834188,-0.9174958026,0.4633973245

O,1.3646528762,1.2443273285,0.5124694645

H,-3.0285460287,0.8670783867,0.136076282

MeSH/CO2 (TS)

Total Energy= -627.283614898

NIMAG= 1

C,-1.5595226952,0.5665364819,0.0724308609

H,-1.4717490641,1.366681309,-0.6705248253

H,-1.1633682704,0.9030109229,1.0363447881

S,-0.6233913526,-0.8948856485,-0.5384694043

H,0.2107790198,-1.2769904163,0.7798493339

C,1.3331938826,-0.1648714419,-0.0457795691

O,1.4103504259,-0.8260179116,1.0310297325

O,1.9258449303,0.614426307,-0.7066801414

H,-2.6107523962,0.2787488274,0.1805338047

MeSCO2H

Total Energy= -627.340304519

NIMAG= 0

C,-1.7680597484,1.0404547995,-0.2982196925

H,-1.317398448,1.400335675,-1.2278927126

H,-1.6559822413,1.7880134193,0.4926650067

S,-0.9699992616,-0.5229630532,0.2110780803

H,1.1490385236,-1.7258563606,0.898492498

C,0.7140361566,0.085267921,0.404949503

O,1.5928966116,-0.8711706948,0.7932004548

O,1.0579751157,1.2206503306,0.2166335116

H,-2.8269717082,0.8051079632,-0.4535646492

(MeSH)2:CO2

Total Energy= -1066.09207331

NIMAG= 0

C,-2.0521747777,1.9626356308,-0.7732379894

H,-2.2717553954,2.4959903284,-1.7051050373

H,-1.3341629593,2.5446078053,-0.186338679

S,-1.3276730454,0.3430249606,-1.2577033785

H,-1.1148184509,-0.1244680103,0.0029551359

C,1.6974400148,1.1016349876,-0.0905633136

O,2.1718043588,0.0803084206,-0.3997179161

O,1.2619665868,2.1326199589,0.2398368199

S,-0.1207807849,-1.7307910419,1.7993142089

H,1.1746097104,-1.3722349084,1.976921273

C,0.1289012449,-2.6843411678,0.2447913634

H,0.7227890897,-3.5841154714,0.4331088581

H,0.6070006853,-2.059912496,-0.5164509387

H,-2.983408618,1.8213945638,-0.2143686059

H,-0.8702036592,-2.9749735603,-0.0983508007

(MeSH)2/CO2 (TS)

Total Energy= -1066.04644954

NIMAG= 1

C,-1.6453046276,0.7221099547,0.0441864344

H,-1.0769434035,1.619963824,-0.2216177721

H,-1.8523643847,0.7092476801,1.1198199509

S,-0.6175669302,-0.7159313597,-0.4285953805

H,-0.6808430757,-1.7184457033,0.9251243096

C,1.1773143699,-0.0332421059,0.2139256113

O,1.8417149591,-0.9627127465,0.738068728

O,1.3537252053,1.1330799367,-0.0140852981

S,-0.0310181352,-2.7008412851,2.0378208645

H,1.1009113747,-1.8766253393,1.4471472643

C,0.1073776645,-4.1071508404,0.857915232

H,1.0194153073,-4.669256267,1.0807199754

H,0.1473287638,-3.7261251658,-0.1690481832

H,-2.5797155487,0.6787908276,-0.5242364

H,-0.7673505691,-4.75291267,0.9823691035

MeSH:MeSCO2H

Total Energy= -1066.08668993

NIMAG= 0

C,-1.562556295,2.0790216865,-1.5159239363

H,-1.0994097652,1.9361618283,-2.4968131627

H,-1.3187524121,3.0720582957,-1.1269637538

S,-0.9760079444,0.7963791973,-0.3541856219

H,-1.2217141665,-1.5240036851,1.7869165015

C,0.7950124119,1.1914923477,-0.3926874235

O,1.5514855171,0.3922243691,0.3699674919

O,1.2504604888,2.0937453317,-1.046960793

S,0.0027962073,-2.0966914318,1.6806513198

H,1.0240212316,-0.3074558923,0.8250847823

C,-0.2652830194,-2.907413773,0.0488645593

H,0.7002180619,-3.338174663,-0.2379675776

H,-0.5737101495,-2.1671768863,-0.6961368171

H,-2.6492785628,1.9541618297,-1.5856918267

H,-1.0078466036,-3.7064375544,0.138156258
